# The influence of the COVID-19 public health emergency and dental coverage on the utilization of endodontic treatment in the United States

**DOI:** 10.3389/fpubh.2026.1783085

**Published:** 2026-04-15

**Authors:** Lorel E. Burns, Carla Shoff, Natalia I. Chalmers

**Affiliations:** 1 Department of Endodontics, New York University College of Dentistry, New York, NY, United States; 2 Independent Consultant, Baltimore, MD, United States; 3 Overjet San Francisco, San Francisco, CA, United States; 4 University of Maryland School of Dentistry, Baltimore, MD, United States

**Keywords:** access to care, COVID-19, dental care, dental insurance, endodontics, health services research, oral health

## Abstract

**Objectives:**

This study aims to examine the impact of the COVID-19 public health emergency on state-level dental service utilization, focusing on endodontic care; and to understand how benefit design and coverage influence healthcare disparities in oral health.

**Methods:**

The data comes from the 2019–2021 Centers for Medicare & Medicaid Services (CMS) unredacted Transformed Medicaid Statistical Information System (T-MSIS). State-level coverage was collected from state Medicaid program provider manuals. We analyzed service utilization rates overall and by demographic factors such as age, sex, and race/ethnicity. Chi-square tests were used to test for significant differences in the rates across the years and within each group. Clustered-robust standard error models were used to predict the odds of receiving an endodontic service.

**Results:**

The COVID-19 pandemic significantly disrupted dental service utilization across the United States, with a marked decrease in preventive and routine dental care. Utilization of endodontic services experienced less of a decline during the pandemic and a significant increase post-pandemic, compared to other dental services. States with more comprehensive Medicaid dental benefits design had significantly higher rates of endodontic service utilization for children and adults. Among children, increased age, female gender, and non-Hispanic Black race/ ethnicity were significantly associated with lower levels of endodontic service utilization. Among adults, rural residence designation was significantly associated with lower levels of endodontic service utilization.

**Conclusion:**

The study highlights the critical role of benefit design in ensuring dental service utilization, particularly during public health emergencies. Oral health stakeholders must recognize the potential long-term consequences of delayed dental care and remain innovative in providing care under limited circumstances.

## Introduction

Oral health is essential to maintaining overall health, as asserted in two major reports examining trends in oral healthcare delivery in America (1, 2). Maintaining good oral health and the natural dentition is important for proper nutrition (3–5), cognitive function (6), and quality of life (1, 2, 7). Endodontics is the branch of dentistry that deals with the health of the dental pulp and tissues surrounding the tooth’s root. Endodontic treatments work by preventing or eliminating infection of the dental pulp (8). Endodontic treatments eliminate pain and are the only way to maintain natural tooth structures once the dental pulp has been compromised. Endodontic services include the following endodontic treatments: vital pulp therapies, non-surgical root canal therapies (initial and retreatment), regenerative endodontic procedures, apexification, and apicoectomies (surgical retreatment). Dental pain and infection are often treated with endodontic treatments, as endodontic treatments are an alternative to tooth extraction in many of these emergent situations.

Two factors greatly impact the utilization of dental services – coverage and cost (total and cost-sharing or out-of-pocket) (9, 10). The financial barriers to receiving dental care are higher than any other type of healthcare (11, 12) and can contribute to tooth loss. Financial barriers and out-of-pocket dental care expenses are most burdensome for low-income individuals. Thus, dental insurance coverage is fundamental to providing access to dental care. In the United States, 38% of children and 16% of adults had public-payer dental insurance in 2021 provided through Medicaid and the Children’s Health Insurance Program (CHIP) (13). Medicaid is the largest payer for dental services for low-income populations, making coverage policies particularly consequential for health equity. The passage of the Affordable Care Act in 2010 made pediatric oral healthcare services one of ten essential health benefits for health plans as defined at 42 U. S. C. 18,021(b) (1)(A) (14). Dental benefits for children eligible for public-payer insurance are provided through Medicaid and the CHIP. The Early and Periodic Screening, Diagnostic and Treatment (EPSDT) benefit enables Medicaid to cover dental services for all child enrollees, and covered dental services must minimally allow for the relief of pain and infection, the restoration of teeth, and maintenance of dental health (15). In contrast to policies that establish minimum dental service requirements for children, states have flexibility regarding whether they will cover dental services for adults in any capacity. The specific dental treatments to be covered are left to the individual states for children and adults (15). Previous research found state-level variation in public-payer coverage for endodontic treatments (16).

The Coronavirus Disease 2019 Public Health Emergency (COVID-19 PHE) resulted in nationwide closures of dental offices and hesitancy among patients to visit the dentist (17). As such, dental practices experienced significant decreases in service utilization, particularly among patients with public-payer dental insurance (18). The demand for endodontic services also declined, but substantially less so than other specialties of dentistry (17, 18). After dental offices re-opened in 2020, forgone dental care led to severe endodontic needs (17) and post-pandemic demand for endodontic services increased (18, 19).

This study evaluated access to care for endodontic services among Medicaid and CHIP beneficiaries. The aims were to (1) describe variations in endodontic treatment coverage at the state-level and endodontic service utilization among Medicaid and CHIP beneficiaries; (2) evaluate the impact of the COVID-19 PHE on endodontic services utilization among Medicaid and CHIP beneficiaries.

## Materials and methods

This retrospective, cross-sectional study included children and adults under 65 enrolled in Medicaid/CHIP who were not dually eligible for Medicare during the study period (2019–2021). Reporting of this study followed the Strengthening the Reporting of Observational Studies in Epidemiology (STROBE) reporting guidelines, was covered by the Common Rule exemption 45 CFR §46.104(d)(4) (iv) and did not require institutional review board review.

### Data sources

This study utilized multiple data sources. The 2019–2021 Centers for Medicare & Medicaid Services (CMS) unredacted Transformed Medicaid Statistical Information System (T-MSIS) Analytic Files (TAF) and Research Identifiable Files (RIF) were used to identify beneficiary characteristics and dental claims. These files were accessed through the CMS Chronic Conditions Warehouse (20). The Economic Research Service Rural–Urban Commuting Area Codes were used to determine the rural/urban status of the beneficiary’s residence ZIP Code (21). The codes on Dental Procedures and Nomenclature (CDT) were used to identify dental and endodontic services associated with dental visits. State Medicaid Policy and Procedure Manuals and fee schedules from 2021 were examined and used to identify state-level coverage of endodontic services, as indicated by CDT.

### Study population

This study included children and adults under 65 enrolled in Medicaid/CHIP who were not dually eligible for Medicare during the study period (2019–2021). To be included in this study, beneficiaries had to live in a state not assigned as having high concern or unusable data quality, according to the CMS DQ Atlas (22). Five states assigned as having high concern or unusable data related to claims volume and professional services procedure codes were excluded from all analyses: Massachusetts, Minnesota, New Jersey, Rhode Island, and Utah. In addition, for all analyses stratified by race/ethnicity, the following nineteen states were excluded due to high concern or unusable race/ethnicity data quality: Alabama, Arizona, Arkansas, Colorado, Connecticut, District of Columbia, Hawaii, Iowa, Kansas, Louisiana, Maryland, Missouri, Montana, New York, Oregon, South Carolina, Tennessee, West Virginia, Wyoming. In total, 39,436,293 child and 30,710,703 adult beneficiaries were included in the analyses that were not stratified by race and ethnicity and a subset of 27,215,085 child and 20,141,527 adult beneficiaries included in the analyses when stratified by race and ethnicity.

### Variables

The outcome variables in this study are (1) whether the beneficiary had any dental service and; (2) whether the beneficiary had an endodontic service. The codes on Dental Procedures and Nomenclature (CDT) were used to identify the following services in the claims data: any dental service (D0100-D9999) and any endodontic service (D3000-D3999).

Beneficiary age was represented as a categorical variable for both children and adults. In accordance with Medicaid coverage guidelines, beneficiaries were considered to be children until the age of 20. Age categories for children were 0–6 years old, 7–11 years old, and 12–20 years old. Age categories for adults were 21–34 years old, 35–49 years old, and 50–64 years old. The analysis also included beneficiary sex, race/ethnicity, and residence designation.

Coverage for endodontic services was evaluated at the CDT level (D3000-D3999) for children (18 codes) and adults (16 codes) for each state in the United States and the District of Columbia. Endodontic services evaluated included the following endodontic CDTs: pulp capping (D3110, pulp cap- direct; D3120, pulp cap- indirect), Pulpotomy (D3220, therapeutic pulpotomy; D3221, pulpal debridement; D3222 partial pulpotomy for apexogenesis), Endodontic therapy on primary teeth (D3230, pulp therapy- anterior tooth; D3240, pulp therapy- posterior tooth); Root canal therapy (D3310, endodontic therapy- anterior tooth; D3320, endodontic therapy- bicuspid tooth; D3330, endodontic therapy-molar), Endodontic retreatment (D3346, retreatment of previous root canal therapy- anterior; D3347, retreatment of previous root canal therapy- bicuspid; D3348, retreatment of previous root canal therapy- molar), Apicoectomy (D3410, apicoectomy- anterior; D3421, apicoectomy- bicuspid; D3425, apicoectomy- molar), Apexification (D3351-D3352), and Pulpal regeneration (D3355-D3357). Endodontic services for children included those for of the primary and permanent teeth. After comparing coverage for endodontic services across states, categorical variables were created for children and adults. State-level endodontic coverage for children: No coverage, 0 services covered; Limited coverage, 1–10 services covered; Extensive coverage, more than 10 services covered. State-level endodontic coverage for adults: No coverage, 0 services covered; Limited coverage, 1–7 services covered; Extensive coverage, 8 or more services covered. The delineation between “limited” and “extensive” coverage was decided after considering how many endodontic services were routinely covered across states, when coverage was provided, for both children and adults.

### Analysis

For each year of the study period (2019–2021), dental service utilization rates per 1,000 pediatric and adult Medicaid/CHIP beneficiaries and endodontic service utilization rates per 1,000 pediatric and adult Medicaid/CHIP beneficiaries with a dental visit during the calendar year were assessed at the state and national levels. Chi-square tests were used to test for significant differences in the rates across the years and within each group. Clustered-robust standard error models were used to predict the odds of receiving an endodontic service. These models fully address the clustered nature of the data at the state-level. Statistical significance was set at *p* ≤ 0.05; all *p*-values were 2-tailed. Analyses were conducted with SAS Enterprise Guide 7.1 and Stata version 18.0. Mapping was used to depict state-level coverage categories for endodontic services and rates of endodontic service utilization for both children and adults. Maps were generated using ESRI ArcMap Pro (2024). Map colors were selected to be colorblind safe and print friendly, based on color recommendations for cartography by Color Brewer 2.0 (2013).

## Results

**Figure 1:**
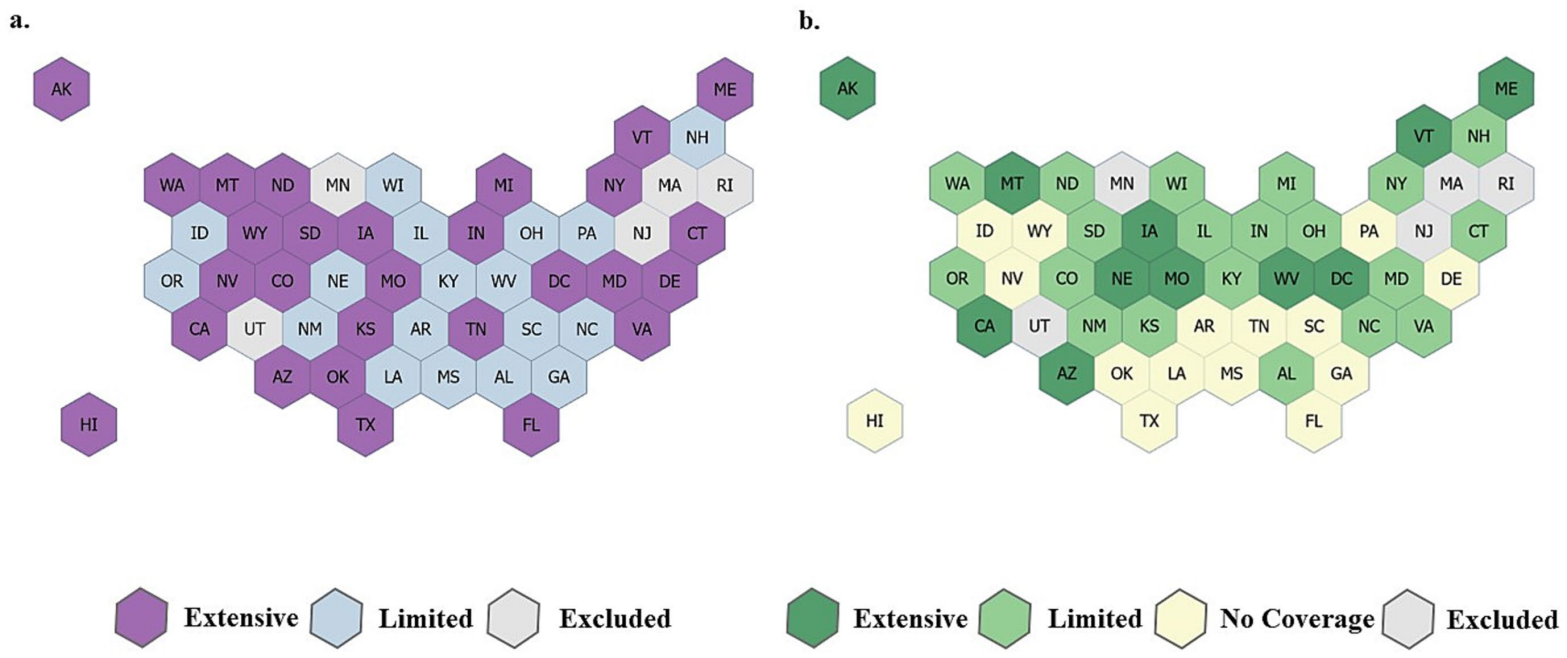
Endodontic treatment coverage maps for adult and children Medicaid beneficiaries. **(a)** Child Medicaid beneficiaries. Extensive coverage, more than 10 procedures covered; limited coverage, 1–10 procedures covered; no coverage, 0 procedures covered; excluded, missing, or unclear state Medicaid policy and procedure manuals and/or fee schedules. **(b)** Adult Medicaid beneficiaries. Extensive coverage, 8 or more procedures covered; limited coverage, 1–7 procedures covered; no coverage, 0 procedures covered; excluded, state Medicaid policy and procedure manuals and/or fee schedules.

For children, all states established payment policies for at least 4 of the 18 endodontic services examined. Some states covered as many as 17 out of 18 of the endodontic services evaluated. For children, all states and the District of Columbia covered therapeutic pulpotomy (CDT D3220) and initial root canal therapy for all tooth types (CDTs D3310, D3320, D3330). The least commonly covered services were those indicated for vital pulp therapy treatments (CDTs D3110, D3120, D3222) and regenerative endodontic treatments (CDTs D3355-D3357). Relevant to the categories of endodontic coverage used in this study, for children, states were categorized as follows: 0 No coverage states (0 services covered); 18 Limited coverage states (1–10 services covered); 28 Extensive coverage (more than 10 services covered); 5 states excluded. In contrast, for adults, 15 states did not provide any endodontic coverage to adult beneficiaries in 2021. The highest levels of endodontic coverage for adults were in states that covered 14 out of 16 of the services evaluated. For adult beneficiaries, states were least likely to cover endodontic services for molar teeth (CDTs D3330, D3348, D3425) and services for vital pulp therapy treatments (CDTs D3110, D3120, D3220). Relevant to the categories of endodontic coverage used in this study, for adults, states were categorized as follows: 15 No coverage states (0 services covered); 20 Limited coverage states (1–7 services covered); 11 Extensive coverage (more than 8 services covered); 5 states excluded. Coverage of endodontic services in 2021 for both children (Figure 1a) and adults exhibited geographic variation (Figure 1b).

**Figure 2:**
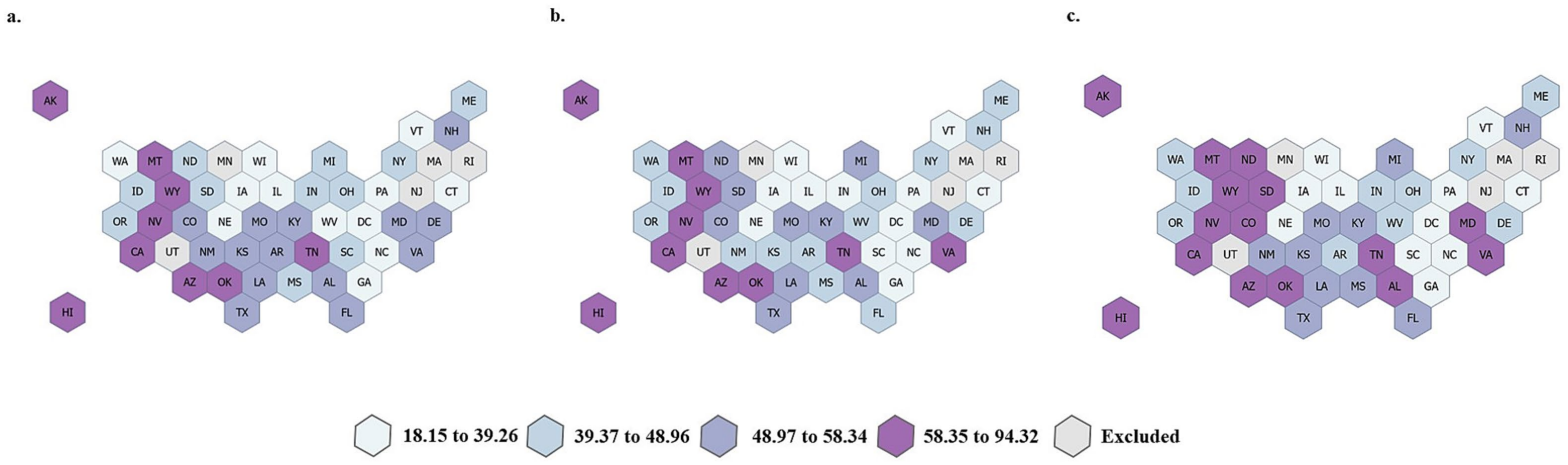
State-level rates of endodontic service utilization per 1,000 Medicaid/CHIP beneficiaries during the study period, children. **(a)** Year, 2019; **(b)** Year, 2020; **(c)** Year, 2021.

**Figure 3:**
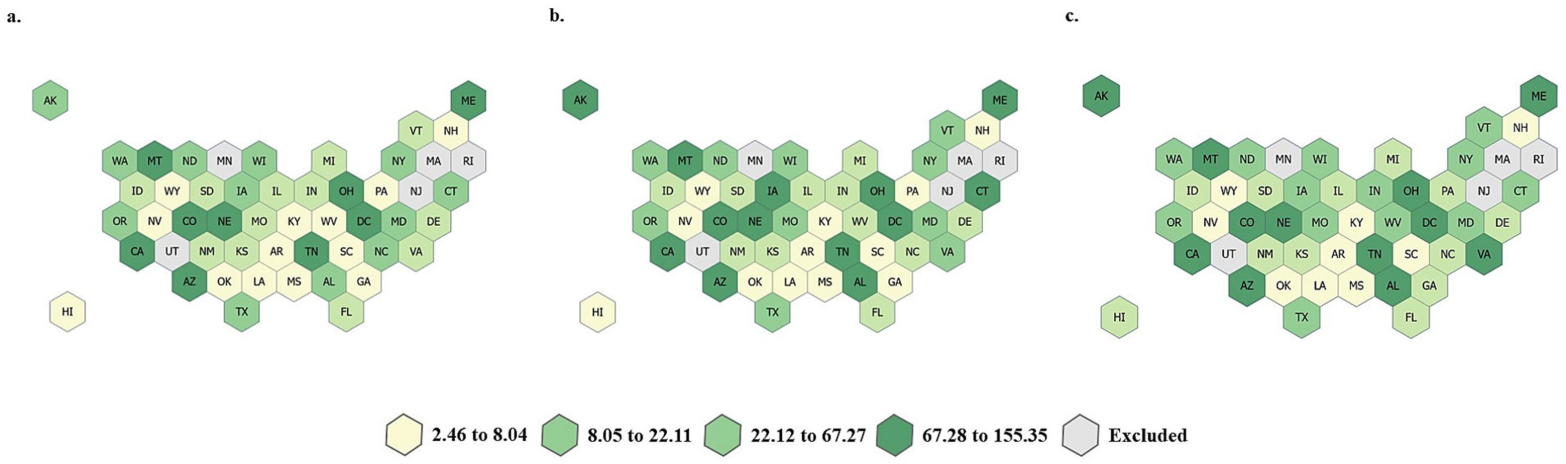
State-level rates of endodontic service utilization per 1,000 Medicaid beneficiaries during the study period, adults. **(a)** Year, 2019; **(b)** Year, 2020; **(c)** Year, 2021.

Table 1 describes the rate of beneficiaries with at least one dental service (D0100-D9999) per 1,000 beneficiaries and the rate of beneficiaries with at least one endodontic service (D3000-D3999) per 1,000 beneficiaries with at least one dental visit for each year of the study (2019–2021) overall and by beneficiary age, sex, race/ethnicity, residence designation (urban/rural), and state-level endodontic coverage. There was a statistically significant decrease in the overall rate of dental service utilization for both children and adults in 2020 compared to 2019. This finding is significant for all age groups, sexes, race/ethnicities, and residence designations. For children and adults who had access to the dental delivery system, endodontic service utilization remained relatively stable during the COVID-19 PHE. For children, a minimal decrease in endodontic service utilization was observed from 2019 to 2020, and for adults, rates of endodontic service utilization remained constant from 2019 to 2020. When comparing rates of utilization pre-COVID-19 PHE (2019) and post-COVID-19 PHE (2021), the rate of utilization of overall dental services rebounded to pre-pandemic levels for children but remained below pre-pandemic rates for adults. In contrast, overall utilization rates for endodontic services were significantly higher in 2021 compared to 2019 for both children and adults. The only demographics for which statistically significant differences in the rates of endodontic service utilization were not observed during the study period were American Indian/ Alaskan Native adults. Rates of endodontic service utilization during the study period are provided at the state-level for both children (Figure 2) and adults (Figure 3). For adults and children, some states deviated from national trends. The following states experienced their lowest rates of endodontic services utilization by adults during the study period, post-PHE (2021): Delaware, Idaho, Illinois, Kansas, New Mexico, Montana, Nebraska, North Carolina, Ohio, Oklahoma, South Dakota, Tennessee, and Washington. For children, the lowest rates of endodontic services utilization were observed during the study period post-PHE (2021) in the following states: Hawaii, Idaho, Ohio, and South Carolina.

**Table 1:** Medicaid/CHIP beneficiaries overall, dental service, and endodontic service utilization counts and rates per 1,000 by year.

	All Beneficiaries			Beneficiaries with dental service utilization			Beneficiaries with endodontic service utilization		
	2019	2020	2021	2019	2020	2021	2019	2020	2021
Beneficiaries Ages 0 to 20 (*N* = 39,436,293)									
Overall	39,436,293	38,107,587	39,255,737	17,482,904	15,309,231	17,439,678	903,747	777,925	937,247
Age groups									
Age 0 to 6	14,081,971	13,203,418	12,987,546	5,400,391	4,515,952	5,059,668	403,067	344,187	406,347
Age 7 to 11	9,887,532	9,524,565	9,764,051	5,456,070	4,735,182	5,334,691	311,632	262,099	324,444
Age 12 to 20	15,466,790	15,379,604	16,504,140	6,626,443	6,058,097	7,045,319	189,048	171,639	206,456
Sex									
Female	19,409,676	18,744,801	19,264,942	8,768,442	7,725,525	8,776,050	443,026	385,953	465,462
Male	20,026,617	19,362,786	19,990,795	8,714,462	7,583,706	8,663,628	460,721	391,972	471,785
Race and ethnicity									
American Indian/Alaskan Native	387,979	350,561	364,032	167,091	126,647	145,712	12,824	8,936	11,030
Asian/Pacific Islander	912,205	870,086	895,956	419,802	349,223	409,222	24,318	19,602	23,420
Non-Hispanic Black	5,473,485	5,343,444	5,443,749	2,174,532	1,917,127	2,181,414	94,548	86,247	101,585
Hispanic	8,468,496	8,130,713	8,332,722	4,279,376	3,703,152	4,098,815	249,046	199,544	237,096
Non-Hispanic White	9,075,230	8,916,802	9,102,981	3,766,586	3,379,648	3,827,466	182,565	164,290	195,017
Multiracial/Other Race/Unknown	2,897,690	3,054,949	3,344,343	1,066,155	1,012,743	1,220,712	57,293	56,713	72,264
Residence designation									
Rural	7,141,818	6,934,993	7,156,063	3,216,721	2,793,998	3,200,023	168,289	143,422	175,033
Urban	32,294,475	31,172,594	32,099,674	14,266,183	12,515,233	14,239,655	735,458	634,503	762,214
State endodontic coverage									
Partial Coverage	13,588,511	13,235,915	13,598,970	6,513,949	5,492,417	6,260,269	265,500	220,543	264,658
Full Coverage	25,847,782	24,871,672	25,656,767	10,968,955	9,816,814	11,179,409	638,247	557,382	672,589
Beneficiaries ages 21 to 64 (*N* = 30,710,703)									
Overall	30,710,703	31,542,645	34,836,000	5,310,618	4,734,258	5,864,575	254,490	226,732	297,617
Age groups									
Age 21 to 34	14,064,004	14,304,052	15,807,152	2,336,026	2,096,561	2,598,368	115,755	103,979	133,004
Age 35 to 49	9,576,509	9,974,886	11,105,547	1,662,698	1,502,060	1,855,842	83,254	75,021	99,063
Age 50 to 64	7,070,190	7,263,707	7,923,301	1,311,894	1,135,637	1,410,365	55,481	47,732	65,550
Sex									
Female	18,503,109	18,958,801	20,818,610	3,464,987	3,087,442	3,812,660	167,164	148,181	192,444
Male	12,207,594	12,583,844	14,017,390	1,845,631	1,646,816	2,051,915	87,326	78,551	105,173
Race and ethnicity									
American Indian/Alaskan Native	299,945	315,433	364,834	50,204	45,029	55,210	2,272	2,018	2,470
Asian/Pacific Islander	1,109,881	1,106,346	1,199,372	208,185	170,319	229,750	12,149	10,022	14,760
Non-Hispanic Black	4,023,389	4,150,549	4,565,251	690,137	641,784	796,402	26,634	24,358	32,999
Hispanic	4,407,932	4,439,229	4,940,491	704,456	623,084	790,428	50,520	43,690	58,964
Non-Hispanic White	8,671,059	9,069,089	9,959,611	1,478,306	1,365,352	1,672,968	61,610	54,809	68,852
Multiracial/Other Race/Unknown	1,629,321	1,774,491	2,004,955	223,914	212,633	275,110	10,906	9,626	14,246
Residence designation									
Rural	5,251,376	5,408,336	5,984,238	841,373	767,286	930,329	28,606	25,957	32,882
Urban	25,459,327	26,134,309	28,851,762	4,469,245	3,966,972	4,934,246	225,884	200,775	264,735
State endodontic coverage									
No Coverage	7,236,772	7,647,474	8,636,017	817,980	806,153	934,494	5,319	6,083	8,394
Partial Coverage	15,114,493	15,565,495	17,135,921	3,152,884	2,759,154	3,440,862	126,016	111,813	147,302
Full Coverage	8,359,438	8,329,676	9,064,062	1,339,754	1,168,951	1,489,219	123,155	108,836	141,921

Overall dental and endodontic service utilization rates per 1,000 Medicaid/CHIP beneficiaries by year								
	Beneficiaries with Dental Service Utilization				Beneficiaries with Endodontic Service Utilization			
	2019	2020	2021	*p*-value	2019	2020	2021	*p*-value
Beneficiaries ages 0 to 20								
Overall	443.3	401.7	444.3	≤0.001	51.7	50.8	53.7	≤0.001
Age groups								
Age 0 to 6	383.5	342.0	389.6	≤0.001	74.6	76.2	80.3	≤0.001
Age 7 to 11	551.8	497.2	546.4	≤0.001	57.1	55.4	60.8	≤0.001
Age 12 to 20	428.4	393.9	426.9	≤0.001	28.5	28.3	29.3	≤0.001
Sex								
Female	451.8	412.1	455.5	≤0.001	50.5	50.0	53.0	≤0.001
Male	435.1	391.7	433.4	≤0.001	52.9	51.7	54.5	≤0.001
Race and ethnicity								
American Indian/Alaskan Native	430.7	361.3	400.3	≤0.001	76.7	70.6	75.7	≤0.001
Asian/Pacific Islander	460.2	401.4	456.7	≤0.001	57.9	56.1	57.2	0.003
Non-Hispanic Black	397.3	358.8	400.7	≤0.001	43.5	45.0	46.6	≤0.001
Hispanic	505.3	455.5	491.9	≤0.001	58.2	53.9	57.8	≤0.001
Non-Hispanic White	415.0	379.0	420.5	≤0.001	48.5	48.6	51.0	≤0.001
Multiracial/Other Race/Unknown	367.9	331.5	365.0	≤0.001	53.7	56.0	59.2	≤0.001
Residence designation								
Rural	450.4	402.9	447.2	≤0.001	52.3	51.3	54.7	≤0.001
Urban	441.8	401.5	443.6	≤0.001	51.6	50.7	53.5	≤0.001
State Endodontic Coverage								
Partial Coverage					40.8	40.2	42.3	≤0.001
Full Coverage					58.2	56.8	60.2	≤0.001
Beneficiaries Ages 21 to 64								
Overall	172.9	150.1	168.3	≤0.001	47.9	47.9	50.7	≤0.001
Age groups								
Age 21 to 34	166.1	146.6	164.4	≤0.001	49.6	49.6	51.2	≤0.001
Age 35 to 49	173.6	150.6	167.1	≤0.001	50.1	49.9	53.4	≤0.001
Age 50 to 64	185.6	156.3	178.0	≤0.001	42.3	42.0	46.5	≤0.001
Sex								
Female	187.3	162.9	183.1	≤0.001	48.2	48.0	50.5	≤0.001
Male	151.2	130.9	146.4	≤0.001	47.3	47.7	51.3	≤0.001
Race and ethnicity								
American Indian/Alaskan Native	167.4	142.8	151.3	≤0.001	45.3	44.8	44.7	0.912
Asian/Pacific Islander	187.6	153.9	191.6	≤0.001	58.4	58.8	64.2	≤0.001
Non-Hispanic Black	171.5	154.6	174.4	≤0.001	38.6	38.0	41.4	≤0.001
Hispanic	159.8	140.4	160.0	≤0.001	71.7	70.1	74.6	≤0.001
Non-Hispanic White	170.5	150.6	168.0	≤0.001	41.7	40.1	41.2	≤0.001
Multiracial/Other Race/Unknown	137.4	119.8	137.2	≤0.001	48.7	45.3	51.8	≤0.001
Residence designation								
Rural	160.2	141.9	155.5	≤0.001	34.0	33.8	35.3	≤0.001
Urban	175.5	151.8	171.0	≤0.001	50.5	50.6	53.7	≤0.001
State endodontic coverage								
No Coverage					6.5	7.5	9.0	≤0.001
Partial Coverage					40.0	40.5	42.8	≤0.001
Full Coverage					91.9	93.1	95.3	≤0.001

Results of regression analyses predicting the odds of a Medicaid beneficiary who had at least one dental visit in 2021 receiving an endodontic service are presented in Table 2. A clustered robust-standard error model for 11,883,341 pediatric beneficiaries shows that compared to beneficiaries aged 0 to 6, beneficiaries aged 7 to 11 were 26.7% less likely to receive an endodontic service, and beneficiaries aged 12 to 20 were 69.5% less likely to receive an endodontic service. Female children were 1.3% less likely to receive an endodontic service compared to male children. Non-Hispanic Black children and multiracial/other/unknown race children were significantly less likely (8.3 and 13.4%, respectively) to receive an endodontic service compared to non-Hispanic white children. American Indian/Alaskan Native children were 37.9% more likely to receive an endodontic service compared to non-Hispanic white children. There were no significant differences in the odds of receiving endodontic services for Asian/Pacific Islander children or Hispanic children when compared to non-Hispanic white children. For children, there is no significant differences in receiving endodontic services across rural and urban areas. Children who live in states with extensive endodontic coverage were 62.3% more likely to receive and endodontic service compared to those who live in states without extensive endodontic coverage.

**Table 2:** Clustered robust-standard error model (CR-SE) predicting the odds of a Medicaid or CHIP beneficiary aged 0 to 64 receiving an endodontic service in 2021.

	Children (*N* = 11,883,341)		Adults (*N* = 3,819,868)
	aOR (95% CI)^a^		aOR (95% CI)^a^
Age groups			
(Ref. Age 0 to 6)		(Ref. Age 21 to 34)	
Age 7 to 11	0.733 (0.647–0.830)^b^	Age 35 to 49	1.099 (0.914–1.322)
Age 12 to 20	0.305 (0.235–0.397)^b^	Age 50 to 64	0.891 (0.658–1.207)
Sex			
(Ref. Male)			
Female	0.987 (0.980–0.994)^b^		1.032 (0.993–1.073)
Race and ethnicity			
(Ref. Non-Hispanic White)			
American Indian/Alaskan Native	1.379 (1.204–1.580)^b^		0.979 (0.665–1.443)
Asian/Pacific Islander	1.061 (0.983–1.146)		0.823 (0.664–1.019)
Non-Hispanic Black	0.917 (0.868–0.968)^b^		1.021 (0.910–1.146)
Hispanic	1.012 (0.759–1.349)		1.009 (0.867–1.174)
Multiracial/Other Race/Unknown	0.866 (0.764–0.982)^b^		0.966 (0.857–1.089)
Residence designation			
(Ref. Urban)			
Rural	1.053 (0.957–1.158)		0.749 (0.599–0.937)^b^
State endodontic coverage			
(Ref. Partial Coverage)		(Ref. No Coverage)	
Extensive Coverage	1.623 (1.261–2.089)^b^	Partial Coverage	3.914 (1.873–8.179)^b^
		Extensive Coverage	11.848 (8.660–16.211)^b^

^a^Models adjusted for all variables in the table, and model is clustered at the state level. The model includes the beneficiaries that live in the 27 states that can be reported because of no claims data or race and ethnicity data quality concerns.

^b^*p* < 0.05.

Results of the clustered robust-standard error model for 3,819,868 adult beneficiaries showed that the most significant predictor of adult beneficiaries receiving an endodontic service in 2021 was state-level endodontic coverage. Adults residing in states with “extensive” endodontic coverage were almost 12 times more likely to have received endodontic services than adults living in states without endodontic coverage. Residence designation (urban/ rural) was the only other significant co-variate; rural beneficiaries were 25.1% less likely to receive an endodontic service compared to their urban counterparts.

## Discussion

The goal of endodontic treatment is to save natural teeth, which ultimately leads to better health outcomes for patients. However, disparities exist in partial and total tooth loss (23) and the utilization of endodontic services (24, 25). Coverage and cost-sharing are two well-known barriers to accessing dental care, including endodontic treatment (11, 26). Medicaid expansions and reform have had positive effects on the accessibility of dental care for children and adults (9, 27, 28). Using a national dataset of Medicaid administrative claims, our study found that state-level coverage for endodontic services was the most significant predictor of utilization. We additionally found that for Medicaid beneficiaries, nationally, the COVID-19 PHE had a lesser impact on the utilization of endodontic services than on dental services overall.

Our study’s findings on national rates of endodontic service utilization during the COVID-19 PHE are consistent with other studies that evaluated the use of dental services during this period (17, 18). To our knowledge, this was the first study that reported variations in endodontic service utilization at the state-level and differences in utilization of endodontic services between children and adults. A minimal decrease in endodontic service utilization was observed for children from 2019 to 2020. For adults, rates of endodontic service utilization remained constant from 2019 to 2020. However, overall utilization rates for endodontic services were significantly higher post-COVID-19 PHE (2021) compared to pre-COVID-19 PHE 2019, for both children and adults nationally. These findings suggest an increase in endodontic disease burden, in part due to deferred dental care, during the COVID-19 PHE (17, 19). Related to the impact of deferred dental care, these findings indicate that telehealth services, which increased in use during the COVID-19 PHE and may have served as an adequate substitute for some in-person dental services, were not sufficient to prevent or address the endodontic needs of the population during this time (29–31). This emphasizes the essential nature of endodontic treatments and endodontic specialists.

Endodontic coverage was the strongest predictor of utilization of endodontic services for both children and adults. For pediatric Medicaid and CHIP beneficiaries, all states covered at least 4 endodontic services. Children residing in states with “extensive” endodontic coverage (more than 10 services covered) were 62.3% more likely to receive an endodontic service. For adults, there was greater state-level variation in endodontic coverage. In 2021, 15 states did not provide any coverage for endodontic services. Adults residing in states with “extensive” endodontic coverage (greater than or equal to 8 services covered) were 11.8 times more likely to receive an endodontic service. For adults, rural residence designation was the only other significant factor predicting the utilization of endodontic services.

Beyond state-level coverage, the utilization of endodontic services for children also varied based on sex, age, race/ethnicity. These observed disparities in endodontic service utilization may reflect differences in need for treatment, differences in motivation to maintain the natural dentition through endodontic treatment, and/ or factors related to access to care beyond dental insurance coverage across demographic groups. Racial and ethnic disparities in dental health, access to care, and use of dental services have persisted in the United States (2, 32, 33). Racial and ethnic disparities observed for children in this national study, are similar to findings of a previous study on the provision and outcomes of endodontic services provided to pediatric Medicaid beneficiaries in a single state (25). For adults, we hypothesize that the extremely large effect of endodontic coverage on the model masked potential demographic disparities in the utilization of endodontic services in this study.

In this study, we evaluated coverage of 18 endodontic services for pediatric beneficiaries and 16 endodontic services for adult beneficiaries. For children, all states and the District of Columbia covered therapeutic pulpotomy (CDT D3220) and initial root canal therapy for all tooth types (CDTs D3310, D3320, D3330). The least commonly covered endodontic services were those indicated for vital pulp therapy treatments (D3110, D3120, D3222) and regenerative endodontic treatments (CDTS D3355- D3357). This is notable because these treatments are associated with some of the most recent biological and clinical advancements in endodontics for the maintenance of natural dentition (34), particularly for pediatric populations (35). For adult beneficiaries, states were least likely to cover endodontic services for molar teeth (CDTs D3330, D3348, D3425) and vital pulp therapy treatments (CDTs D3110, D3120, D3220).

Our analysis has limitations. The primary limitation is one that applies to other analytic studies using administrative claims, it is possible that data were missing, resulting from events that may not be captured in the claims or inaccuracies that may exist in the claims data. Relevant to this study, administrative claims data are not able to capture the need for dental procedures in a population and cannot account for any treatment rendered for which insurance was not billed. In an effort to analyze only the most reliable data available, data from 5 states were excluded from all analyses in this study if determined by CMS to have high concern or unusable data related to claims volume and professional services procedure codes. Further, an additional 19 states, determined by CMS to have high concern or unusable data related to race/ ethnicity, were excluded from regression analyses. The exclusion of this data may affect the generalizability of the disparity results reported in this study. Beyond the limitations of the use of administrative claims data for research, this study did not consider other potentially important barriers to endodontic service utilization beyond insurance coverage. Another measurable barrier that could have been evaluated in this study is provider participation with public payer dental coverage. The omission of this factor may have resulted in the impact of insurance coverage being over-emphasized. Overall, the national dataset of Medicaid administrative claims utilized in this study make the findings relevant to populations of Medicaid beneficiaries across the United States and potentially beneficiaries of public-payer insurance outside of the United States. The findings of this study, however, may not be generalizable to those with private-payer insurance or those without dental insurance.

## Conclusion

Endodontic treatments aim to reduce dental pain, and infection, and maintain the natural dentition, ultimately contributing to overall health. For child and adult Medicaid beneficiaries, state-level endodontic coverage was the strongest predictor of utilization of endodontic services. The COVID-19 Public Health Emergency significantly affected overall dental service utilization nationwide. However, the stability in endodontic utilization throughout this period underscores its essential role in dental care.

## Data Availability

The data analyzed in this study is subject to the following licenses/restrictions: The 2019–2021 Centers for Medicare & Medicaid Services (CMS) unredacted Transformed Medicaid Statistical Information System (T-MSIS) Analytic Files (TAF) and Research Identifiable Files (RIF) were used to identify beneficiary characteristics and dental claims. These files were accessed through the CMS Chronic Conditions Warehouse. Requests to access these datasets should be directed to https://www.medicaid.gov/medicaid/data-systems/macbis/medicaid-chip-research-files/transformed-medicaid-statistical-information-system-t-msis-analytic-files-taf.

## References

[1] U.S. Department of Health and Human Services. Oral Health in America: A Report of the Surgeon General. Rockville, MD: National Institutes of Health, National Institute of Dental and Craniofacial Research (2000).

[2] U.S. Department of Health and Human Services. Oral Health in America: Advances and Challenges. Bethesda, MD: National Institutes of Health, National Institute of Dental and Craniofacial Research (2021). 35020293

[3] Kikutani T Yoshida M Enoki H Yamashita Y Akifusa S Shimazaki Y . Relationship between nutrition status and dental occlusion in community-dwelling frail elderly people. Geriatr Gerontol Int. (2013) 13:50–4. doi: 10.1111/j.1447-0594.2012.00855.x 22489562

[4] Toniazzo MP Amorim PS Muniz F Weidlich P. Relationship of nutritional status and oral health in elderly: systematic review with meta-analysis. Clin Nutr. (2018) 37:824–30. doi: 10.1016/j.clnu.2017.03.014, 28392164

[5] Ma KS Chan SY Van Dyke TE Wang SI Wei JC Ashina S. Tooth loss and chronic pain: a population-based analysis of the National Health and nutrition examination survey. J Pain. (2024) 25:104529. doi: 10.1016/j.jpain.2024.104529 38588761

[6] Nakamura H Noguchi-Shinohara M Ishimiya-Jokaji M Kobayashi Y Isa M Ide K . Brain atrophy in normal older adult links tooth loss and diet changes to future cognitive decline. NPJ Aging. (2024) 10:20. doi: 10.1038/s41514-024-00146-4, 38519528 PMC10960014

[7] Sanders AE Slade GD Lim S Reisine ST. Impact of oral disease on quality of life in the US and Australian populations. Community Dent Oral Epidemiol. (2009) 37:171–81. doi: 10.1111/j.1600-0528.2008.00457.x, 19175659 PMC3760707

[8] National Commission on Recognition of Dental Specialties and Certifying Boards. Specialty definitions. Endodontics. (2021). Available online at: https://ncrdscb.ada.org/en/dental-specialties/specialty-definitions (Accessed October 1, 2024)

[9] Decker SL Lipton BJ. Do Medicaid benefit expansions have teeth? The effect of Medicaid adult dental coverage on the use of dental services and oral health. J Health Econ. (2015) 44:212–25. doi: 10.1016/j.jhealeco.2015.08.009, 26519908 PMC6758545

[10] Singhal A Damiano P Sabik L. Medicaid adult dental benefits increase use of dental care, but impact of expansion on dental services use was mixed. Health Aff (Millwood). (2017) 36:723–32. doi: 10.1377/hlthaff.2016.0877, 28373339

[11] Vujicic M Buchmueller T Klein R. Dental care presents the highest level of financial barriers, compared to other types of health care services. Health Aff (Millwood). (2016) 35:2176–82. doi: 10.1377/hlthaff.2016.0800, 27920304

[12] Centers for Disease Control and Prevention, National Center for Health Statistics National Health Interview Survey (2023)

[13] Agency for Healthcare Research and Quality Medical expenditure panel survey - household full year consolidated data files (2002-2021) (2023) 38416859

[14] Centers for Medicare & Medicaid Services Information on essential health benefits (EHB) benchmark plans (2024) Available online at: https://www.cms.gov/marketplace/resources/data/essential-health-benefits (Accessed November 5, 2024).

[15] Centers for Medicare & Medicaid Services Dental Care: Medicaid.gov (2023) Available online at: https://www.medicaid.gov/medicaid/benefits/dental-care/index.html (Accessed November 5, 2024).

[16] Burns LE Gencerliler N Terlizzi K Solis-Roman C Sigurdsson A Gold HT. Apexification outcomes in the United States: a retrospective cohort study. J Endodont. (2023) 49:1269–75. doi: 10.1016/j.joen.2023.07.020, 37517583 PMC10543604

[17] Choi SE Mo E Sima C Wu H Thakkar-Samtani M Tranby EP . Impact of COVID-19 on dental care utilization and oral health conditions in the United States. JDR Clin Transl Res. (2024) 9:256–64. doi: 10.1177/23800844231165016, 37082861 PMC10125887

[18] Choi SE Simon L Basu S Barrow JR. Changes in dental care use patterns due to COVID-19 among insured patients in the United States. J Am Dent Assoc. (2021) 152:1033–43.e3. doi: 10.1016/j.adaj.2021.07.002, 34656295 PMC8444228

[19] Nosrat A Yu P Dianat O Verma P Taheri S Wu D . Endodontic specialists' practice during the coronavirus disease 2019 pandemic: 1 year after the initial outbreak. J Endodont. (2022) 48:699–706. doi: 10.1016/j.joen.2022.03.004, 35307515 PMC8928705

[20] Centers for Medicare & Medicaid Services, Chronic Conditions Data Warehouse. (2023). Available online at: https://www2.ccwdata.org/web/guest/home/ (Accessed June 7, 2023)

[21] Economic Research Service Rural-urban commuting area codes (2003)

[22] Centers for Medicare & Medicaid Services, Medicaid and CHIP Buisness Information Solutions. DQ Atlas. (2023). Available online at: https://www.medicaid.gov/dq-atlas/welcome. (Accessed November 5, 2024).

[23] Fleming E Afful J Griffin SO. Prevalence of tooth loss among older adults: United States, 2015-2018. NCHS Data Brief. (2020) 368:1–8. 32600520

[24] Burns LE Gencerliler N Gold HT. A comparative analysis of public and private dental benefit payer types for the provision and outcomes of root canal therapy on permanent teeth of children and adolescents in Massachusetts. J Am Dent Assoc. (2023) 154:151–8. doi: 10.1016/j.adaj.2022.10.011, 36528395 PMC10026184

[25] Burns LE Gencerliler N Terlizzi K Wu Y Solis-Roman C Gold HT. A comparative analysis of outcomes of root canal therapy for pediatric medicaid beneficiaries from New York state. Front Oral Health. (2022) 3:1031443. doi: 10.3389/froh.2022.1031443, 36479449 PMC9720667

[26] Burns LE Gencerliler N Ribitzki U Yashpal S Feldman L Sigurdsson A . Access to care considerations for the endodontic treatment of immature permanent teeth: a National Survey of pediatric dentists and endodontists. J Endodont. (2024) 50:1100–7. doi: 10.1016/j.joen.2024.05.009, 38796057

[27] Kenney GM Marton J Klein AE Pelletier JE Talbert J. The effects of Medicaid and CHIP policy changes on receipt of preventive care among children. Health Serv Res. (2011) 46:298–318. doi: 10.1111/j.1475-6773.2010.01199.x, 21054374 PMC3037784

[28] Wehby GL Lyu W Shane DM. The impact of the ACA Medicaid expansions on dental visits by dental coverage generosity and dentist supply. Med Care. (2019) 57:781–7. doi: 10.1097/MLR.0000000000001181, 31433313 PMC6742569

[29] Monaghesh E Hajizadeh A. The role of telehealth during COVID-19 outbreak: a systematic review based on current evidence. BMC Public Health. (2020) 20:1193. doi: 10.1186/s12889-020-09301-4, 32738884 PMC7395209

[30] Martinho FC Griffin IL. A cross-sectional survey on the impact of coronavirus disease 2019 on the clinical practice of endodontists across the United States. J Endodont. (2021) 47:28–38. doi: 10.1016/j.joen.2020.10.002, 33058936 PMC7550122

[31] Nosrat A Dianat O Verma P Yu P Wu D Fouad AF. Endodontics specialists' practice during the initial outbreak of coronavirus disease 2019. J Endodont. (2022) 48:102–8. doi: 10.1016/j.joen.2021.09.015, 34626613 PMC8493639

[32] Flores G Tomany-Korman SC. Racial and ethnic disparities in medical and dental health, access to care, and use of services in US children. Pediatrics. (2008) 121:e286–98. doi: 10.1542/peds.2007-1243, 18195000

[33] Lee JY Divaris K. The ethical imperative of addressing oral health disparities: a unifying framework. J Dent Res. (2014) 93:224–30. doi: 10.1177/0022034513511821, 24189268 PMC3929974

[34] Murray PE Garcia-Godoy F Hargreaves KM. Regenerative endodontics: a review of current status and a call for action. J Endodont. (2007) 33:377–90. doi: 10.1016/j.joen.2006.09.013, 17368324

[35] Hargreaves KM Giesler T Henry M Wang Y. Regeneration potential of the young permanent tooth: what does the future hold? J Endodont. (2008) 34:S51. doi: 10.1016/j.joen.2008.02.032 18565373

